# Repurposing Laboratory Plastic into Functional Fibrous Scaffolds via Green Electrospinning for Cell Culture and Tissue Engineering Applications

**DOI:** 10.1021/acsbiomaterials.5c00146

**Published:** 2025-04-10

**Authors:** Nael Berri, Sandhya Moise, Antonios Keirouz, Andrew Jennings, Bernardo Castro-Dominguez, Hannah S. Leese

**Affiliations:** † Department of Chemical Engineering, 1555 University of Bath, Bath BA2 7AY U.K.; ‡ Department of Mechanical Engineering, University of Bath, Bath BA2 7AY U.K.; § Centre for Bioengineering and Biomedical Technologies (CBio), University of Bath, Bath BA2 7AY U.K.; ∥ Centre for Digital Manufacturing and Design (dMaDe), University of Bath, Bath BA2 7AY U.K.

**Keywords:** green solvent, electrospinning, biomaterials, tissue engineering, cell-scaffold interaction, sustainable tissue engineering

## Abstract

Cell culture for tissue engineering is a global and flexible research method that relies heavily on plastic consumables, which generates millions of tons of plastic waste annually. Here, we develop an innovative sustainable method for scaffold production by repurposing spent tissue culture polystyrene into biocompatible microfiber scaffolds, while using environmentally friendly solvents. Our new green electrospinning approach utilizes two green, biodegradable and low-toxicity solvents, dihydrolevoglucosenone (Cyrene) and dimethyl carbonate (DMC) to process laboratory cell culture petri dishes into polymer dopes for electrospinning. Scaffolds produced from these spinning dopes, produced both aligned and non-aligned microfiber configurations, were examined in detail. The scaffolds exhibited mechanical properties comparable to cancellous bones whereby aligned scaffolds achieved an ultimate tensile strength (UTS) of 4.58 ± 0.34 MPa and a Young’s modulus of 11.87 ± 0.54 MPa, while the non-aligned scaffolds exhibited a UTS of 4.27 ± 0.92 MPa and a Young’s modulus of 20.37 ± 4.85. To evaluate their potential for cell-culture, MG63 osteoblast-like cells were seeded onto aligned and non-aligned scaffolds to assess their biocompatibility, cell adhesion, and differentiation, where the cell viability, DNA content, and proliferation were monitored over 14 days. DNA quantification demonstrated an eight-fold increase from 0.195 μg/mL (day 1) to 1.55 μg/mL (day 14), with a significant rise in cell metabolic activity over 7 days, and no observed cytotoxic effects. Confocal microscopy revealed elongated cell alignment on aligned fiber scaffolds, while rounded, disoriented cells were observed on non-aligned fiber scaffolds. Alizarin Red staining and calcium quantification confirmed osteogenic differentiation, as evidenced by mineral deposition on the scaffolds. This research therefore demonstrates the feasibility of this new method to repurpose laboratory polystyrene waste into sustainable cell culture tissue engineering scaffolds using eco-friendly solvents. Such an approach provides a route for cell culture for tissue engineering related activities to transition towards more sustainable and environmentally conscious scientific practices, thereby aligning with the principles of a circular economy.

## Graphical abstract

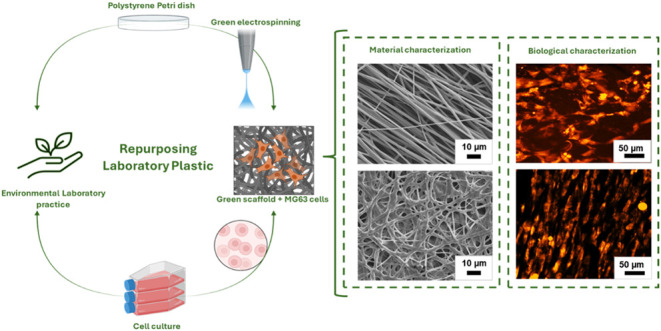

## 1 Introduction

Plastic waste is one of the most pressing environmental challenges, contributing significantly to global warming and climate change throughout its life cycle.^1–3^ Efforts on both global and national scales are addressing this issue including alternative methods and practices within research and development laboratories.^4,5^ Cell culture and tissue engineering laboratories also contribute significantly to plastic waste, with polystyrene (PS) being one of the most popular materials due to its transparency, biocompatibility, and ease of sterilization.^6^ Items such as plastic petri dishes or cell culture-well plates, often discarded after a single use or upon expiration, are a major waste source. It is estimated that scientific laboratories worldwide generate over 5.5 million metric tons of plastic waste annually, with over 20,500 research institutions contributing to the total.^7^ Such figures highlight the importance of adopting sustainable practices, including upcycling waste into valuable resources through nontoxic and recyclable processes and reducing the environmental footprint of laboratory operations. By targeting polystyrene waste, there is an opportunity to promote a circular economy in research while addressing the broader challenges of plastic waste and climate change.^8,9^

Among the range of available laboratory techniques, electrospinning is widely used to create high-surface-area fibrous scaffolds for tissue engineering, mimicking both hard and soft tissue.^10,11^ The process involves using a high-voltage differential between a spinneret and a collector to generate fibers from a polymeric solution, allowing researchers to tailor materials for applications like osteochondral tissue regeneration through controlled optimization of fiber diameters and morphologies.^12^

However, electrospinning’s reliance on toxic solvents such as dimethylformamide (DMF), tetrahydrofuran (THF), and chloroform poses significant health and environmental concerns.^13^ These solvents are highly effective at dissolving polymers but pose significant health risks to researchers due to their toxicity and volatility. Their environmental implications are also significant, as specialized disposal methods are required to mitigate their hazardous effects, increasing the overall cost and ecological burden of electrospinning-based research. From a regulatory perspective, the use of conventional solvents such as THF is increasingly regulated due to safety and environmental concerns.^14–16^ It has driven researchers to explore safer, more sustainable alternatives, particularly green solvents. These solvents are characterized by their lower toxicity, reduced environmental impact, and compatibility with renewable resources.^17^ They have been used in various scientific applications as alternatives to traditional organic solvents.^17,18^ Acetone, acetic acid, and formic acid have been used as low-toxicity alternatives in electrospinning, but they introduce other risks, such as high flammability, corrosiveness, and volatility, which can compromise laboratory safety and contribute to air pollution. These drawbacks restrict their broader adoption in electrospinning.^19,20^ Moreover, research on green solvents in electrospinning has primarily focused on natural polymers like silk and cellulose derivatives, with synthetic plastics like polystyrene remaining underexplored (studies conducted between 2020 and 2024 reveal that only a small number of publications have specifically investigated electrospinning and green solvents, Figure S1).

In this study, it is hypothesized that integrating polystyrene waste with green solvents which include dihydrolevoglucosenone (Cyrene) and dimethyl carbonate (DMC) can overcome these limitations and enable the creation of sustainable biomaterial scaffolds via electrospinning. Cyrene and DMC, derived from renewable resources, are biodegradable and offer low-toxicity alternatives to traditional solvents.^21^ While these solvents have been used individually in various applications, such as green solvent replacements and pharmaceutical precursors, their combination in electrospinning remains unexplored, presenting unique technical challenges.^23–27^ This dual approach presents unique technical challenges, such as lower polymer solubility, variations in evaporation rates, and impacts on solution viscosity and fiber formation, requiring careful optimization of electrospinning parameters to ensure scaffold quality and functionality. To address these challenges, this study explores in detail the potential of combining Cyrene and DMC as green solvents for upcycling polystyrene waste from expired tissue culture plastics. By processing polystyrene under milder conditions, these solvents reduce energy consumption and eliminate the need for hazardous catalysts typically required in conventional recycling processes. Traditional recycling methods, such as catalytic degradation, often involve high temperatures and energy demands, contributing to a larger carbon footprint,^22–24^ while enzymatic degradation, although safer, suffers from slow reaction rates and limited enzyme availability.^25^ In contrast, leveraging the unique properties of Cyrene and DMC to produce functional scaffolds through an environmentally benign process, bridges the gap between sustainable materials science and biomedical innovation. To investigate the influence of scaffold structure on functionality, both aligned and nonaligned fibers were fabricated and studied. Aligned fibers mimic the anisotropic structure of native bone tissue, providing directional cues for osteoblast elongation and enhanced mechanical strength. Nonaligned fibers, in contrast, offer isotropic structures with higher porosity, promoting cell attachment and proliferation. MG63 cells, a well-characterized osteoblast-like cell line, were chosen for their reproducibility, consistent behavior, and osteogenic potential, making them ideal for evaluating scaffold performance. The study examines how variations in fiber alignment affect mechanical properties and osteoblast responses, offering insights into the optimization of scaffolds for bone tissue engineering.

By successfully integrating green solvents with the upcycling of polystyrene waste and optimizing scaffold architecture, this study addresses critical challenges in both environmental sustainability and tissue engineering. The new approach outlined here aims to reduce the environmental impact of laboratory operations while advancing the field of biomaterials, providing a pathway to create eco-friendly scaffolds for biomedical applications.

## 2 Experimental Section

### 2.1 Materials

Dihydrolevoglucosenone (Cyrene, ≥98.5%) and dimethyl carbonate (DMC, 99%) were sourced from Sigma-Aldrich. Polystyrene (PS) with an average molecular weight of 273 kg mol^–1^ (Figure S2), used as the polymer in the electrospinning solution, was derived from Petri dishes supplied by Fisher Scientific. The supplied Petri dishes were additive-free to ensure compatibility with cellular studies, as additives could exhibit cytotoxic properties. Petri dishes were processed into fine particles using a RETSCH MM 400 Mixer Mill (RETSCH GmbH, Haan, Germany) at a frequency of 26 Hz for 2 min and 30 s (Figure S3). Deionized water was prepared using a Purelab Chorus 1 Complete water purification system (Elga LabWater, Veolia Water Systems LTD). All materials were used as received without further purification.

### 2.2 Scaffold Fabrication

The scaffolds were fabricated using an electrospinning setup designed to produce uniform microfibers for cell culture applications. Recycled PS powder, derived from ground Petri dishes, was dissolved in a green solvent mixture of DMC and Cyrene at a 75:25 ratio (v/v) to prepare a 15% (w/v) polymer solution (Figure 1A). The dissolution process was carried out at 70 °C with continuous magnetic stirring for 8 h until a homogeneous polymer solution was achieved, exhibiting a viscosity of 316 ± 9 mPa·s (mean ± SD, *n* = 3) measured at a shear rate of 100 s^–1^. Viscosity measurements were performed at 25 °C using an Anton Paar MCR 72 rheometer equipped with a parallel-plate geometry. Approximately 2 mL of polymer solution was carefully loaded onto the measuring plate, ensuring a uniform sample layer and avoiding air bubble entrapment. Electrospinning was conducted under controlled environmental conditions, maintaining a relative humidity of 35–45% and a temperature range of 28–30 °C to ensure fiber uniformity. The polymer solution was dispensed at a constant flow rate of 1 mL/h using a syringe pump (AL-1000, World Precision Instruments, Sarasota, FL) through a 20-gauge blunt needle with an inner diameter of 0.6 mm. The needle-to-collector distance was set at 15 cm (Figure 1A). A voltage of +12 kV was applied to the needle, with −2 kV applied to the collector to generate the electric field required for fiber formation. The electrospinning process ran continuously for 5 h.

**Figure 1:**
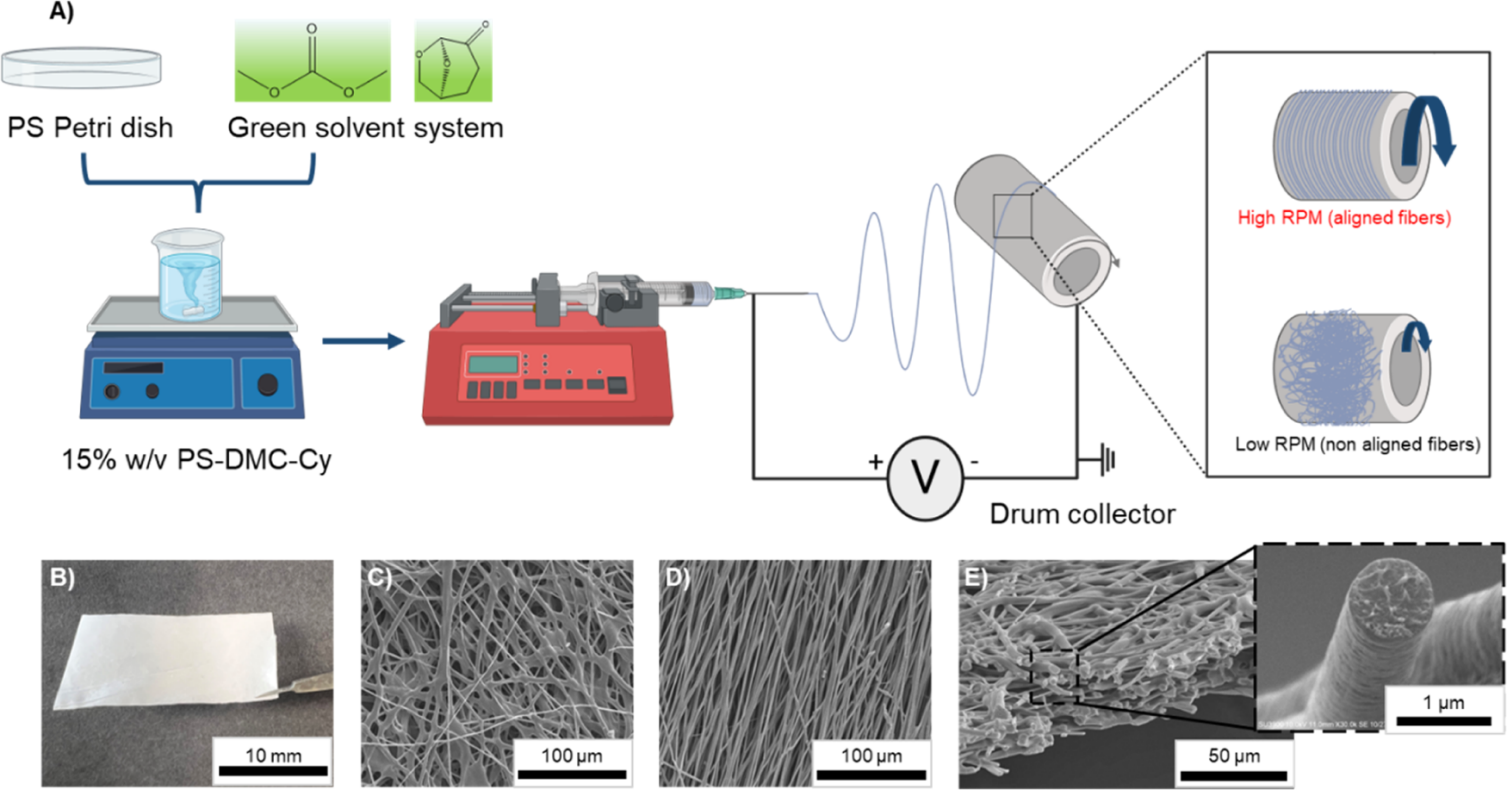
(A) Schematic diagram illustrating the experimental procedure for fabricating polystyrene electrospun scaffolds using a green solvent and recycled Petri dish material, with fibers collected onto a rotating drum collector. (B) Photograph of the fabricated scaffold. (C) SEM micrograph of the scaffold showing nonaligned fibers. (D) SEM micrograph of the scaffold showing aligned fibers. (E) Cross-sectional SEM image of the scaffold, with a zoomed-in view highlighting the morphology of a single fiber.

To fabricate aligned fibers, a rotating drum collector (diameter: 89 mm; width: 200 mm) with a high rotational speed of 1800 rpm was used, while nonaligned fibers were collected at a lower rotational speed of 200 rpm which is effectively equivalent to no rotation in terms of fiber alignment.^26,27^ The resulting scaffold appears as a white, homogeneous structure (Figure 1B). Depending on the rotation speed during electrospinning, the scaffold consists of either nonaligned fibers (Figure 1C) or aligned fibers (Figure 1D). The cross-sectional view (Figure 1E) reveals a uniform scaffold architecture with well-defined, single round fibers. To facilitate scaffold removal, the collector’s surface was covered with aluminum foil during fabrication. Following electrospinning, the scaffold was immersed in distilled water for 2 h and then left to dry overnight under a fume hood to ensure the removal of any residual solvent. These preparation steps yielded scaffolds with distinct morphologies, optimized for following cell culture studies.

To assess the feasibility of recycling used Petri dishes as a polymer source, pristine dishes were first immersed in MG63 cell culture media for 4 days to simulate realistic biological contamination typical in terms of mechanical and biocompatible properties of laboratory conditions. Following incubation, the dishes were sterilized using ethanol treatment combined with 30 min of UV exposure and then processed identically to noncontaminated control samples to produce electrospun fibers.

### 2.3 Fiber Morphology Scaffold Characterization

The fiber diameter and alignment of scaffolds were characterized using scanning electron microscopy (SEM) with a Hitachi SU3900. Samples were vacuum-dried for at least 24 h and coated with a 20 nm gold layer to ensure image clarity. Micrographs were captured from three distinct areas of each scaffold at magnifications ranging from 100 to 10,000×. The membrane thickness was measured using a micrometer caliper for all samples used in characterization and cell studies, and these measurements were further verified through SEM imaging.

For fiber diameter analysis, three micrographs from three aligned samples and three nonaligned samples were processed using a Python-based pipeline. The code automated the measurement and comparison of fiber diameters in aligned and nonaligned scaffolds. It detected scale bars to calculate a scale factor for converting pixel measurements to microns, applied thresholding and segmentation to isolate fibers, and used spatial filtering to ensure measurements were well-distributed and nonoverlapping. Exactly, 400 measurements per image were processed, and statistical analysis was performed on the filtered diameters. Results were visualized through annotated images (Figure S4) and detailed histograms. The pipeline utilized the PoreSpy library,^28^ adapted to treat voids between fibers as pseudopores, enabling efficient extraction of spatial and size-related metrics. Features like dynamic scale detection, spatial filtering, and precise binning ensured accurate, reproducible, and efficient data analysis. Additionally, the orientation of fibers was analyzed using a computational method known as structure tensor analysis to assess how consistently the fibers are aligned. SEM images were first converted to grayscale to ensure uniform processing. The method calculated the direction of fibers, their alignment consistency (coherence), and the strength of features in the image. A smoothing factor (σ = 2) was applied to reduce noise while emphasizing key structures. Fiber orientation was determined by identifying the dominant direction in each region, and coherence measured how well the fibers aligned in a single direction, with higher coherence values indicating better alignment. To visualize the results, rose plots (showing the distribution of orientation angles), probability density plots, and color-coded maps were generated.

### 2.4 Material Scaffold Characterization

Fourier transform infrared (FTIR) spectroscopy was performed to identify functional groups, assess structural changes due to interactions with the green solvents, and detect any residual solvent in the scaffolds. The FTIR spectra were collected using a Nicolet iS 5 infrared spectrometer equipped with the iD7 attenuated total reflectance (ATR) module (Thermo Fisher Scientific). Data acquisition was performed over a wavenumber range of 4000–500 cm^–1^, averaging 126 scans per spectrum with a spectral resolution of 1 cm^–1^. The water contact angle was measured using the sessile drop technique with a high-speed camera (Krüss GmbH). A 10 μL drop of distilled water was pipetted onto the scaffold’s top surface, and static contact angles were calculated as the mean ± SD of ten replicates using the DataPhysics OCA 25 with the SCA 20 software module (Figure S5). The scaffolds underwent plasma treatment using a Diener Electronic plasma-surface system. The treatment was performed for 1 min at a pressure of 1 mbar and a power of 100 W to modify the surface properties, improving cell attachment by overcoming the hydrophobic nature of the scaffolds (Figure S5).

The molecular weights of different materials were determined using gel permeation chromatography (GPC) on an Agilent Infinity 1200 system equipped with two Polymer Laboratories (Agilent) Mixed D columns and a guard column, maintained at 35 °C in a column oven. The system featured a multidetector suite, including dual-angle light scattering, a viscometer, and a refractometer, operating in THF at a flow rate of 1 mL/min. Calibration was performed using linear narrow molecular weight standards of polystyrene and poly­(methyl methacrylate), with molecular weights ranging from 580 g/mol to 280,000 g/mol.

The pore size and distribution of the scaffolds were determined using a gas–liquid displacement technique with a POROLUX 1000 porometer (POROMETER nv, Belgium). The scaffolds were initially saturated with POREFIL, a specialized wetting liquid provided by the manufacturer. Nitrogen gas (N_2_) was subsequently introduced to displace the wetting liquid from the pores. The pressure of N_2_ was progressively increased, and the resulting flow of gas through the newly opened pores was measured. At each pressure level, the system was stabilized for 2 s before recording the pressure and flow data, ensuring accurate and consistent results. The pore size corresponding to each pressure was calculated using the Young–Laplace eq (eq 1)­

1 $$d=\frac{4\gamma \cos\theta }{\Delta P}$$

Where *d* is the pore diameter, γ is the surface tension of the wetting liquid (16 mN/m), θ is the contact angle of the wetting liquid on the membrane surface, and Δ is the pressure difference across the membrane.

The tensile properties of the scaffold were evaluated using MFS tensile testing machine equipped with a 2 N load cell. Scaffold samples were cut into rectangular strips (3 × 1 mm^2^) and mounted in the testing machine using pneumatic grips to prevent slippage. All samples were visually confirmed to break clearly within the gauge region, away from the grips, validating that the measured tensile properties accurately reflect scaffold performance. Samples that did not fracture within the gauge region were excluded from the analysis. The test was conducted at room temperature under a controlled displacement rate of 5 mm/min, ensuring consistent strain application. All measurements were performed on at least 6 replicates to ensure reproducibility. Differential scanning calorimetry (DSC) was performed using a TA Instruments Q20 under nitrogen (50 mL/min). Approximately 5–10 mg of scaffold sample was sealed in an aluminum pan. The sample was equilibrated at −20 °C for 5 min, then heated from −20 to 150 °C at 10 °C/min. Upon reaching 150 °C, it was immediately cooled back down to −20 °C at 10 °C/min. Thermogravimetric analysis (TGA) was performed using a NETZSCH STA 449F1 analyzer with an alumina (Al_2_O_3_) crucible under a nitrogen atmosphere (100 mL/min). Three repeats of each sample (∼6.3 mg) were heated from 30 to 800 °C at 10 °C/min. Thermogravimetric (TG) curves, derivative thermogravimetric (DTG) curves, and differential scanning calorimetry (DSC) curves were recorded and analyzed using NETZSCH Proteus software to assess thermal stability, decomposition behavior, and residual solvent content.

### 2.5 Cell Culture

The electrospun scaffolds were cut into 1 × 1 cm^2^ films and sterilized by soaking in 70% ethanol for 1h followed by exposure to ultraviolet light.

MG63 cells were cultured at 37 °C, 95% humidity, and 5% CO_2_ in expansion media which consisted of Dulbecco’s Modified Eagle’s Medium (DMEM) supplemented with 10% fetal bovine serum (FBS), 1% penicillin-streptomycin solution and 1% glutaMAX. Cells were seeded onto the surface of electrospun biomaterial scaffolds placed in flat-bottomed Costar Ultra-Low Attachment 24-Well Plates (Corning, U.K.). Cells were seeded at 7000 cells·cm^–2^ onto the scaffold surfaces, left undisturbed for a few hours (to promote cell attachment) before adding expansion medium, which was subsequently changed every 2 days. In this study, phosphate-buffered saline (PBS, Gibco, pH 7.4) was used as a standard buffer solution for material preparation and cell culture application. Cellular viability, proliferation and osteogenic differentiation studies

Presto Blue assay was adopted to assess the metabolic activity of the MG63 cells seeded on the green electrospun scaffolds (aligned fibers and non-aligned fibers) after 1, 3, 5, and 7 days of culture. A 10% Presto Blue solution (diluted in culture media) was added to the samples and incubated at 37 °C for 40 min. The fluorescence was measured at 530 nm excitation and 590 nm emission to assess cell viability via a Biotek Synergy HT plate reader (BioTek Instruments, Winooski, VT). In addition, Pico green was used to estimate the number of cells on a longer 14-day period exploring the long-lasting effect of the scaffold on the cells. The assay was conducted by adding a 1X PicoGreen reagent solution to the samples, incubating for 40 min in the dark, and measuring fluorescence at 480 nm excitation and 520 nm emission via the same card reader. The controls were MG63 seeded on a cell culture flat plastic surface that were passaged every 48 h.

MG63 cells were seeded onto scaffolds as mentioned previously and cultured in expansion media for 4 days. Once cells reached approximately 80% confluence, the expansion medium was replaced with osteogenic medium. Osteogenic media consisted of the expansion media further supplemented with 0.1 μM dexamethasone, 50 μM ascorbic acid, and 50 mM β-glycerophosphate. The cells were cultured for 21 days, with media exchange every 3 days once, to promote osteogenic differentiation.

### 2.6 Cell Morphology and Fluorescence Staining

To evaluate cell morphology and adhesion on scaffolds, live-cell imaging was conducted over 48 h using a Zeiss Cell Discovery 7 confocal microscope. CellTracker Orange CMRA dye (Fisher Scientific) was prepared at a working concentration of 25 μM in prewarmed culture media. The prepared working solution was added to cells seeded on scaffolds and incubated for 60 min under optimal growth conditions (37 °C, 5% CO_2_, and 95% relative humidity). After incubation, the dye solution was carefully removed and replaced with fresh expansion media to maintain cell viability. Confocal microscopy was used to capture live-cell images at specified intervals throughout the observation period. Imaging was performed with an excitation wavelength of 557 nm and emission detection at 572 nm to ensure optimal fluorescence signal. The procedure enabled real-time visualization of cell morphology, distribution, and interactions with the scaffold surface. The scaffolds were visualized at their original thickness (100 μm) using a custom-designed 3D-printed holder, specifically created for this work. The holder had a diameter of 1.54 cm and a visualization window of 0.8 cm^2^, allowing media to flow from inside the holder to the outside while preventing the scaffolds from shifting as the microscope moved between positions (Figure S6).

### 2.7 Alizarin Red Staining and Quantification

To assess differentiation, MG63 cells were stained for calcium using the Alizarin Red stain on days 1, 7, 14, and 21. The cells were fixed with 4% paraformaldehyde for 10–15 min at room temperature and stained using a 2% Alizarin Red S solution (pH 4) with excess dye washed off by PBS rinses. To quantify the Alizarin Red staining bound to calcium, the dye was dissolved using 10% acetic acid added to each sample and incubated with gentle shaking at room temperature for 30 min to ensure complete dye extraction. Each sample had three replicates. A 150 μL aliquot from each sample was transferred to a new plate, and absorbance measured at 450 nm. Calcium concentration was analyzed daily across six groups (Table 1).

**Table 1:** Definition of Groups for Alizarin Red Staining and Quantification

group name	description
AF exp	cells cultured on aligned fiber scaffold expansion media
no-AF exp	cells cultured on nonaligned fiber scaffold in expansion media
AF ost diff	cells cultured on an aligned fiber scaffold in osteogenic media
no-AF ost diff	cells cultured on a nonaligned fiber scaffold in osteogenic media
control exp	cells cultured without a scaffold in expansion media
control ost diff	cells cultured without a scaffold in osteogenic media

Baseline correction was carried out by subtracting the mean absorbance of blank samples from all measurements. Calcium concentration (μg/mL) was then calculated using a conversion factor obtained from a calibration curve, which was generated by plotting absorbance values against known calcium concentrations stained with Alizarin Red S. For each day, mean and standard deviation were calculated for each group, and Tukey’s Honest Significant Difference (HSD) posthoc test was used to evaluate pairwise differences in calcium concentration (*p* < 0.05). Significance brackets were assigned to groups based on the Tukey HSD results, with brackets indicating significant differences between groups. Data were visualized with bar plots showing mean calcium concentrations and standard deviations, with significance letters annotated to indicate statistical relationships. Statistical analyses and visualizations were performed in Python using pandas, scikit, posthocs, and matplotlib.

## 3 Results and Discussion

### 3.1 Green Solvent Polymer Solubilization

In preparation for electrospinning, the polymer must first be dissolved, and general solubility data reviewed to select appropriate solvents. The main challenge in dissolving polystyrene is its highly nonpolar and hydrophobic nature, which creates a solubility mismatch with most typically used green solvents.^29^ Several solvents, including 2-methyl tetrahydrofuran (2-MeTHF), triethyl phosphate, and DMSO, were tested for their ability to dissolve spent tissue culture polystyrene^30^ (Table S1). While many solvents showed limited effectiveness, a combination of dimethyl carbonate (DMC) and Cyrene successfully dissolved polystyrene.

Cyrene, with its Hansen solubility parameters—dispersion (δ_D_), polar (δ_P_), and hydrogen bonding (δ_H_)—does not align well with the nonpolar characteristics of polystyrene. Specifically, polystyrene is hydrophobic and nonpolar, whereas the relatively high polarity and hydrogen bonding capacity of Cyrene limits its ability to dissolve it.^31^ However, Cyrene exhibits unique solvation properties due to its bicyclic structure containing two adjacent cyclic ether groups, which impart hydrotropic characteristics, enhancing polymer solubility.^32^ DMC, a polar protic solvent, also faces challenges in dissolving polystyrene due to its polarity, however, the success of combining Cyrene and DMC in dissolving polystyrene can be attributed to two key mechanisms. First, improved solubility parameter matching: when used together, Cyrene and DMC form a solvent system with intermediate polarity, making it more suitable for dissolving polystyrene than either solvent used individually. A second plausible mechanism is that the combination of Cyrene and DMC improves the solvent mixture’s ability to penetrate and diffuse into the polymer matrix. Increased diffusion reduces the restrictions posed by polymer entanglement, making it easier for the solvent mixture to penetrate and solubilize polystyrene, particularly in the case of high-molecular-weight polystyrene.^29^

Molecular weight analysis demonstrated that the green solvent system preserved the polymer’s molecular integrity during dissolution and electrospinning (Figure S2). Metrics such as weight-average molecular weight (*M*_w_), number-average molecular weight (*M*_n_), and polydispersity index (PDI) were consistent across all samples: Purified polystyrene (286,231 g/mol), solubilized polystyrene (281,718 g/mol), green scaffold fibers (269,907 g/mol), and lab Petri dish (276,424 g/mol). The observed molecular weight distribution of the green scaffold, characterized by a relatively narrow polydispersity index (PDI) of 2.51, suggests minimal polymer degradation, cross-linking, or aggregation during processing. This consistency in molecular weight distribution highlights the ability of the preparation method in maintaining polymer integrity. As expected, the molecular weight of solubilized polystyrene in the green scaffold, Petri dish polystyrene, and electrospun fibers remained unchanged, confirming that the green solvent and electrospinning process did not degrade or alter the polymer’s molecular structure. This outcome validates the assumption that the observed differences in fiber properties are due solely to changes in molecular arrangement and morphology, rather than any chemical modifications to the polymer.

In the dRI signal vs retention time graph, the observed dRI signal is influenced by both the polymer concentration and the refractive index increment (d*n*/d*c*), which reflects how much the polymer alters the refractive index of the solution. Since d*n*/d*c* is constant for the same polymer–solvent system, the lower dRI signals for purified and solubilized polystyrene compared to the other samples can be attributed to reduced polymer concentration. This reduction is likely due to residual solvent diluting the samples, rather than any change in the intrinsic properties of the polymer. Despite these variations, the retention of high molecular weight fractions and stable molecular weight metrics demonstrated the compatibility of the Cyrene-DMC solvent system with polystyrene. These findings confirm that the solvent system effectively dissolves polystyrene Petri dishes without compromising molecular stability, supporting the use of lab Petri dish-derived polystyrene as a reliable source material for electrospun fibers with consistent mechanical and morphological properties.

The FTIR spectra show that the polystyrene electrospun fibers (from the Petri dish), polystyrene pellets, and unprocessed polystyrene Petri dish exhibit the same characteristic peaks (Figure 2A). These include the range of 3080–2850 cm^–1^, corresponding to C–H stretching vibrations, where 3080 cm^–1^ is attributed to aromatic C–H stretching from the benzene rings, and 2920 and 2850 cm^–1^ are associated with aliphatic C–H stretching from the polymer backbone. Additional peaks are observed at 1600 and 1490 cm^–1^, corresponding to C = C stretching in the aromatic ring, 1450 cm^–1^ for C–H bending, and 761 and 701 cm^–1^ for C–H out-of-plane bending vibrations.^33^ The consistent appearance of these peaks across all samples confirms no significant chemical changes to the polystyrene backbone even after mixing with Cyrene and DMC solvent or during the electrospinning process for scaffold fabrication. The FTIR complements the evidence provided by GPC, demonstrating the preservation of the polymer’s molecular structure with no significant chemical modifications.

**Figure 2:**
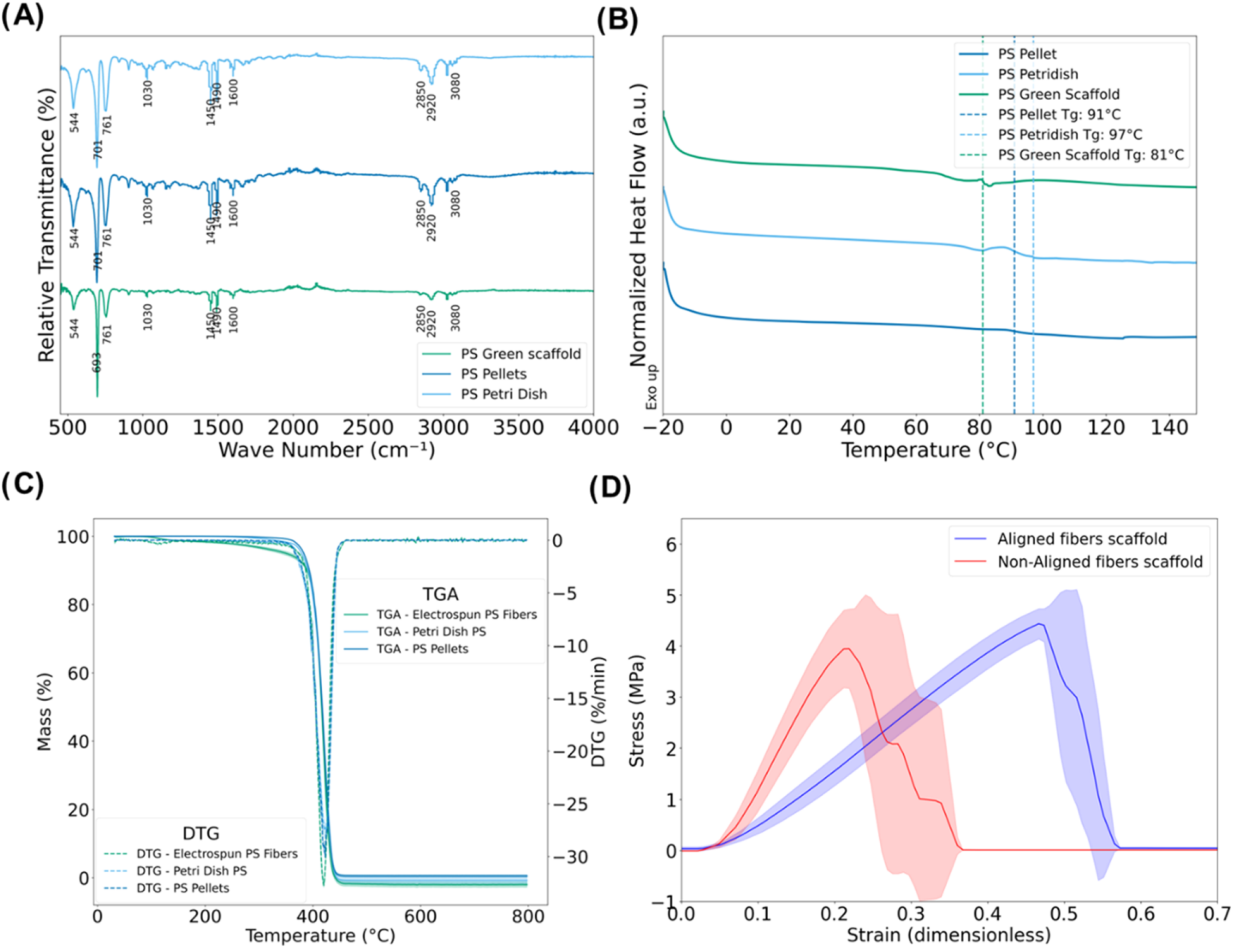
Comparative analysis of polystyrene sources and scaffold properties:(A) FTIR spectra of PS from recycled Petri dishes, pellets, PS bulk material, and green scaffolds (B) DSC characterization of fibers and bulk materials (C) TGA and DTG thermal stability: (D) Stress–strain behavior of aligned vs nonaligned fiber scaffolds (mean ± SD).

The aim of the DSC analysis was to evaluate the effect of solubilization and electrospinning on the physical and thermal properties of polystyrene (PS), specifically its glass transition temperature (*T*_g_), as an indirect measure of polymer solubility and structural changes. DSC was performed on the electrospun PS scaffold, PS pellet (unprocessed), and PS Petri dish (Figure 2B). The glass transition temperature (*T*_g_) of the PS Petri dish (97 °C) and PS Pellet (91 °C) were the highest, reflecting their more rigid and crystalline structure.^34^ This higher *T*_g_ is consistent with the unprocessed state of the PS Petri dish, which has tightly packed, crystalline polymer chains, typical of a rigid, solid form. In contrast, the PS green scaffold exhibited a lower *T*_g_ of 81 °C, showing greater polymer chain flexibility and reduced crystallinity compared to the PS Petri dish and PS Pellet.^35^ This reduction in *T*_g_ suggests that the solubilization and electrospinning processes disrupted the polymers ordered structure, making the scaffold less rigid and easier to transition from a glassy to a rubbery state. The more flexible, amorphous nature of the scaffold confirms that solubilization was successful, as the polymer chains became less tightly packed and more mobile.^36^ Additionally, the porous structure introduces voids or “free volume” within the polymer matrix, which can enhance chain mobility and lower *T*_g_ value.^37^ To determine whether the *T*_g_ reduction was due to residual solvent plasticization or structural changes, TGA and DTG analyses were performed. No significant mass loss below 200 °C was observed in any sample, confirming the absence of substantial residual solvent. However, a very small mass loss in the DTG of the electrospun fibers at approximately 100 °C suggests the presence of minimal residual solvent. Additionally, the gradient of the TGA curve from around 300 °C differs for the electrospun fibers compared to the bulk polymer, likely due to the increased surface area of the fibers. The overlapping TGA decomposition profiles and similar DTG peak temperatures (∼400 °C) across all samples further indicate that the primary factor contributing to the *T*_g_ reduction in electrospun fibers is morphological changes—such as increased surface area, higher free volume, and reduced crystallinity—rather than solvent-induced plasticization.^38^

Further insights into these structural changes are provided by the mechanical performance of aligned and nonaligned Petri-dish derived polystyrene electrospun fibers produced using green solvents, which demonstrates distinct differences due to fiber orientation and the influence of the solvents on fiber structure (Figure 2C). Aligned fibers exhibit a UTS of 4.58 ± 0.30 MPa and a strain-to-failure of 0.65 ± 0.03, while nonaligned fibers show a UTS of 4.27 ± 0.92 MPa and a strain-to-failure of 0.31 ± 0.05. The standard deviation of the UTS for nonaligned fibers (0.92 MPa) was significantly higher than that of aligned fibers (0.30 MPa), as illustrated by the wider shaded region in the stress–strain curves for nonaligned fibers, which reflects their greater variability. Similarly, the standard deviation of strain-to-failure was higher for nonaligned fibers (0.05) compared to aligned fibers (0.03), reflecting inconsistent deformation behavior in the former group (Figure S7). While the variability in strain-to-failure is not directly represented by the shadowing on the stress–strain curves, the wider shaded region for nonaligned fibers reflects greater variability in stress values at corresponding strain points, further emphasizing the inconsistent mechanical performance of nonaligned fibers (Figure 2D and Table S2). Compared to other electrospun materials reported in the literature (Table 2), the green scaffold possess UTS values similar to smooth PS fibers (2.78 MPa)^39^ and wrinkled PS fibers (7.76 MPa)^39^ while maintaining a Young’s Modulus of 11.87–20.37 MPa, within the range of soft/hyaline cartilage (4–12 MPa) and synthetic scaffolds like polylactic acid (PLA: 7.5–15 MPa)^40,41^ and polycaprolactone (PCL: 3–190 MPa).^42^ However, it aligns well with native tissue requirements and synthetic scaffolds designed for osteogenesis differentiation and mineralization.^41,42^

**Table 2:** Mechanical Properties of the Green Scaffold Compared with Synthetic Electrospun Scaffolds and Native Cartilage (This Study Concentrates on Studies with Similar Fiber Diameters, Thicknesses, and Alignments to Achieve a Meaningful Comparison)

material/scaffold type	UTS (MPa)	Young’s modulus (MPa)	references
green scaffold (aligned fibers)	4.58 ± 0.34	11.87 ± 0.54	present work
green scaffold (nonaligned fibers)	4.27 ± 0.92	20.37 ± 4.85	present work
soft/hyaline cartilage	3.5–9	4–12	^45^
electrospun PLA scaffold (various applications)	2–15	7.5–15	^40,41,46^
electrospun PCL scaffold for bone regeneration	1–5	3–190	^42^
electrospun PS (various applications)	0.40–7.76	16.25–127.9	^39,47,48^

The lower Young’s Modulus of the green scaffold compared to published work on electrospun polystyrene fibers (e.g., smooth PS: 127.9 MPa)^44,46,47^ can be attributed to the use of green solvents during fabrication. Cyrene (boiling point 227 °C) has higher boiling points and can result in slower evaporation during electrospinning.^43^ The slower evaporation may cause fibers to retain residual solvent which can reduce molecular alignment and crystallinity and ultimately lower the scaffold’s stiffness.^44^ Nonetheless, the use of green solvents remains a sustainable and functional alternative for tissue engineering, balancing eco-friendliness with mechanical properties suitable for applications in bone regeneration and tissue repair.

Additional data were collected using a biologically contaminated polystyrene (PS) Petri dish. Electrospun scaffolds made from media-exposed PS showed a reduced glass transition temperature (*T*_g_) of 61 °C, compared to 81 °C for those from non-biocontaminated PS (Figure S8). Given PS Petri dish chemical stability, significant degradation from 4 days of media contact is unlikely. Instead, the *T*_g_ shift could be due to small internal changes—such as increased free volume or altered chain packing—introduced during reprocessing.

TGA and DTG analyses of clean fibers, when compared with those of media-exposed fibers (Figure S9), show similar decomposition profiles: the primary degradation occurs around 400 °C (with an onset near 300 °C). This result indicates that 4 days of media contact does not alter the fiber scaffold thermal stability, despite a modest reduction in *T*_g_. Furthermore, SEM analysis (Figure S10) confirmed that media exposure did not change fiber morphology (average diameter: 2.9  ±  1.22 μm), ruling out residual solvent effects. Overall, these results support that repurposed polystyrene Petri dishes, even following biological contact, can be repurposed into electrospun fibrous scaffolds utilizing green solvents, for cell culture and tissue engineering. Further investigations are required to understand molecular-level effects and long-term performance in cell culture.

### 3.2 Aligned and Nonaligned Fiber Morphology

During optimization, trials utilizing lower concentrations of polystyrene within the green solvent system consistently produced beads rather than fibers (as shown in Figure S11), underscoring the critical role of polymer concentration in attaining the desired scaffold structure. When compared to the literature, the polymer concentration (10–40% w/v), voltage (12–18 kV), flow rate, and collector distance (approximately 20 cm) used in this study fall within the typical ranges reported, indicating that with the correct ratio of green solvents, it is feasible to produce sustainable PS fiber.^48,49^

Key features for tissue engineering scaffolds are fiber diameter, alignment, and porosity.^50^ SEM imaging showed that the optimized fibers in both scaffolds were smooth, well-rounded, and free of beads, with cross-sectional images revealing uniform, circular shapes (Figure 3A,B). The average fiber diameters were controlled, measuring 2.35 μm (SD = 1.4 μm) for nonaligned fibers and 2.0 μm (SD = 1.2 μm) for aligned fibers, with a significant difference in average size between aligned and nonaligned scaffolds (*p*-value = 1.3 × 10^–8^).

**Figure 3:**
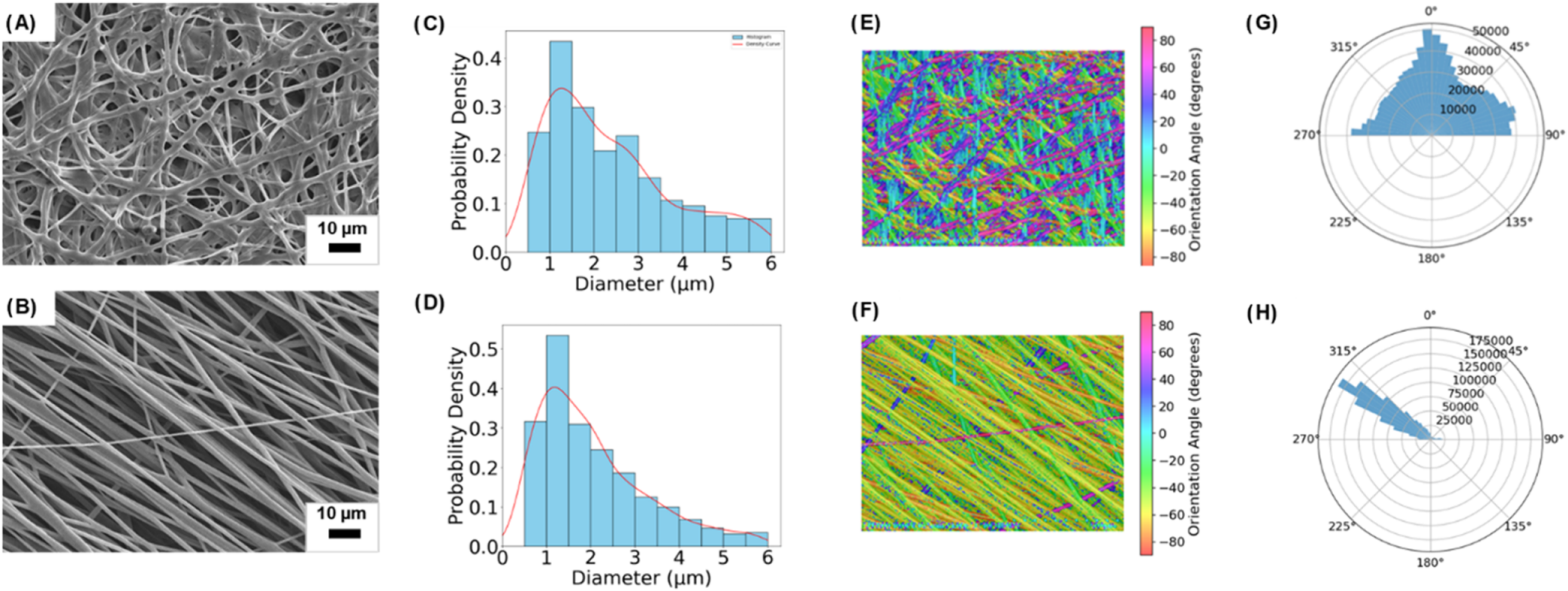
Comprehensive analysis of fiber morphology and orientation in PS green scaffolds: SEM images showcasing (A) nonaligned and (B) aligned fibers with 400 diameter measurements per sample, Probability density distributions of fiber diameters for (C) nonaligned and (D) aligned fibers. Fiber orientation visualizations highlighting (E) nonaligned and (F) aligned fibers. Rose plots displaying orientation distributions for (G) nonaligned and (H) aligned fibers.

For the nonaligned scaffold, diameter distribution analysis revealed a right-skewed pattern with most diameters in the 1–2 μm range (Figure 3C), and a few reaching values above, contributing to a more heterogeneous scaffold structure. This anisotropic deposition is likely due to the fibers crossing at various angles and overlapping contributing to a complex heterogeneous network. In contrast, the aligned scaffold displayed a more concentrated distribution around the 1–1.5 μm range, with fewer fibers extending above 3 μm (Figure 3D). Tighter control of fiber size in the aligned scaffold, achieved using a rotating drum collector, enhances its uniform surface topography and enhanced stretching forces by imparting a tangential velocity to the deposition surface during deposition.

Two limitations were observed, including, fibers positioned very close to each other and partially adhered during the process before drying can lead to the formation of larger fibers, thereby increasing the standard deviation and contributing to scaffold inhomogeneity. Second, fibers not lying on the same plane exhibit variations in diameter measurements, further adding to the overall variability in fiber size. These morphological differences between aligned and nonaligned fibers influence their mechanical properties and interactions with cells. Aligned fibers promote directional cell growth and organization, while nonaligned fibers are reported to provide a more isotropic environment suitable for nondirectional tissue applications such as tendons or ligament as they more closely mimic the tissues’ viscoelastic properties.^51,52^

Using structure tensor analysis, fiber orientation and alignment were quantitatively assessed for both samples. The analysis provided metrics such as average orientation, orientation standard deviation, and coherence, enabling a detailed evaluation of fiber alignment. These findings were visually confirmed through the rose plot and color-coded orientation map, highlighting the distinct alignment patterns between the samples (Figure 3E,F). As expected, nonaligned fibers exhibited high variability in fiber orientation (SD: 52°), indicating a random, isotropic distribution, while aligned fibers showed lower variability (SD: 39°) and stronger directional alignment (Figure 3G,H). Fiber alignment is anticipated to influence cellular organization, while the nonaligned scaffold’s random orientation supports diverse cellular interactions in applications that do not require directional cues.^53^ Porosity measurements (using POROLUX 1000) showed similar values for both scaffold types, averaging 7.4 μm, supporting adequate nutrient and oxygen diffusion for cell viability (Figure S12). These results highlight how fiber diameter and alignment can be effectively controlled in green solvent electrospun scaffolds, demonstrating that upcycled materials like polystyrene can achieve high-quality, functional biomaterials.

### 3.3 Cell Behavior on Sustainable Scaffolds

Cells cultured on the scaffolds showed a gradual increase in estimated cell numbers, as measured by PrestoBlue at specific time points at different time points (Figure 4A). Normalized results indicated that the estimated cell numbers on the nonaligned scaffolds were comparable to those observed on the positive control (2D flat surface of a 24-well plate). Statistical analysis revealed no significant differences between the nonaligned fibers and the control at most time points, except on day 7, when the control displayed a significantly higher cell number than the nonaligned fibers (*p* = 0.00631). Additionally, by day 7, a significant difference emerged between the aligned and nonaligned fibers (*p* = 0.03661), with the aligned fibers exhibiting the highest estimated cell numbers. Remarkably, the aligned fibers consistently supported cell growth comparable to or exceeding that of the control, particularly at day 7, despite being fabricated from upcycled polystyrene.

**Figure 4:**
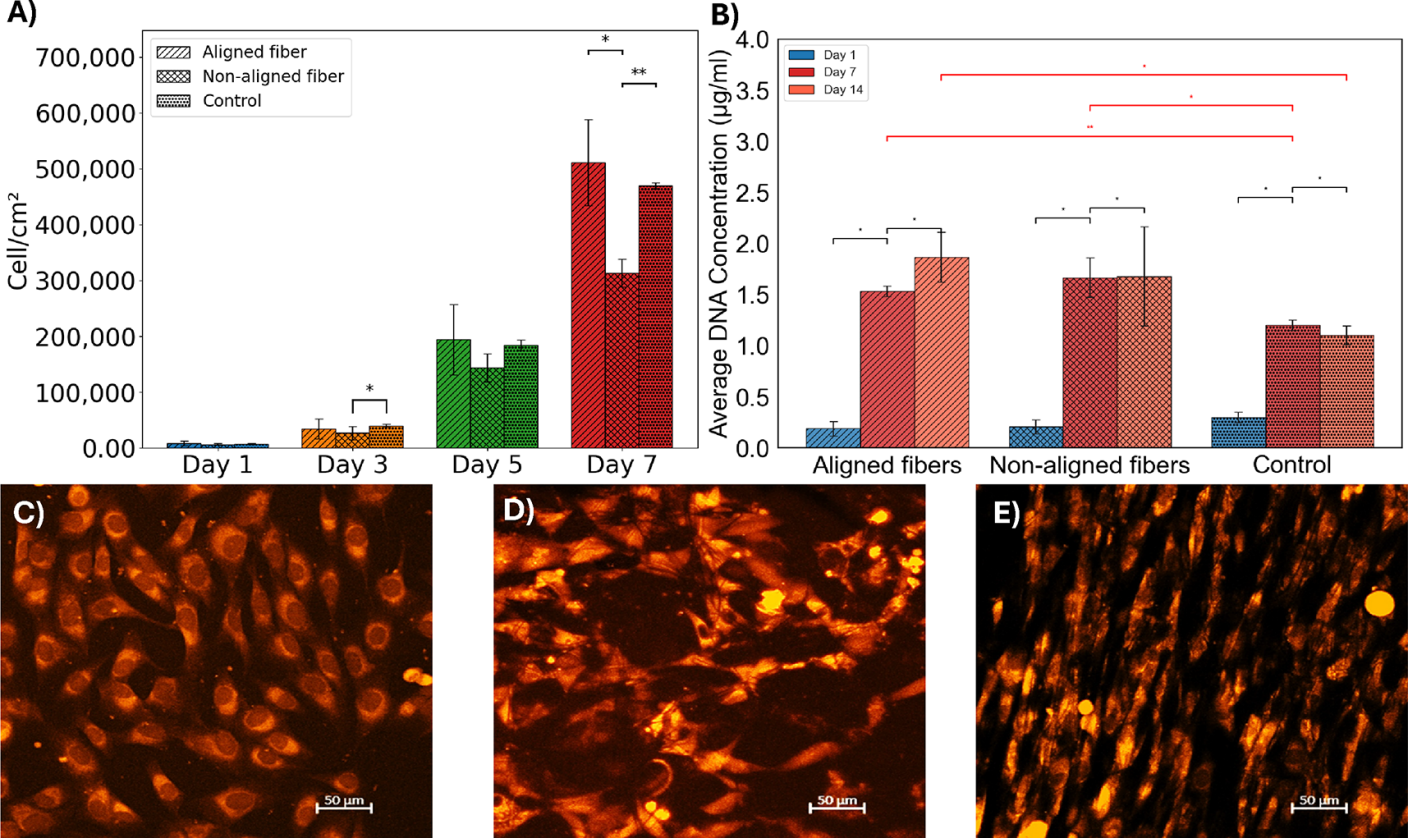
Evaluation of MG63 cell behavior seeded on aligned and nonaligned fiber scaffolds and control (A) MG63 cell density obtained via PrestoBlue over 7 days, highlighting cell viability and proliferation trends. (B) DNA concentration quantified via PicoGreen assay at days 0, 7, and 14, with black brackets showing within-group comparisons across days and red brackets standing for within-day comparisons across groups. asterisks denote statistical significance (*p < 0.05, **p < 0.01, ***p < 0.001). Confocal images (cell tracker staining) on day 7 illustrate cell morphology and distribution for (C) control, (D) nonaligned fibers, and (E) aligned fibers.

These findings are noteworthy as they demonstrate that scaffolds made from upcycled polystyrene can perform as well as, or even better than, conventional cell culture plates. The results align with existing literature, which highlights the role of fiber alignment in providing directional cues that enhance cell elongation, migration, and organization along the fiber axis.^51,52,54^ The observed behavior of the aligned fibers reinforces their suitability for tissue engineering applications, supporting their potential as an effective alternative in this field.

Seeding efficiency was further evaluated through DNA quantification over a 14-day period (Figure 4B). DNA concentration in the scaffold samples increased from 0.195 μg/mL on day 1 to approximately 1.55 μg/mL by day 14. DNA concentrations in both aligned and nonaligned scaffold samples increased from 0.195 μg/mL on day 1 to approximately 1.55 μg/mL by day 14. In contrast, the control group reached a lower DNA concentration of 1.24 μg/mL by day 14.

Notably, the fluorescence signals from the PrestoBlue assay were converted into “estimated cell numbers” by referencing a standard curve and normalizing to the scaffold’s surface area. While this method offers an accurate indirect estimate of cell density per cm^2^, it primarily reflects cellular metabolic activity, which can vary across different culture conditions. As such, higher normalized fluorescence values may indicate an increased total cell count, enhanced per-cell metabolic activity, or a combination of both. In line with these findings, the control showed fewer total cells (based on DNA quantification) but relatively high fluorescence, suggesting a robust metabolic activity per cell. In contrast, the aligned fiber scaffolds supported significantly higher cell numbers and displayed strong Fluorescence, indicating both effective proliferation and sustained metabolic function. Interestingly, while nonaligned fibers showed higher DNA content than the control, their cell estimation were slightly lower, implying that cells on nonaligned fibers might be more numerous yet exhibit comparatively lower per-cell metabolic rates. Overall, these results emphasize that upcycled polystyrene scaffolds, particularly those with aligned fibers, not only support higher cell numbers (as indicated by DNA quantification) but can also promote strong metabolic activity (as reflected by PrestoBlue fluorescence measurements). The 2D control, while sustaining fewer cells, remains metabolically robust on a per-cell basis, highlighting how culture environment influences both cell proliferation and function.

To gain deeper insight into the behavior of cells on the scaffolds, confocal microscopy images (Figure 4C–E) were used to examine cell morphology and distribution.^59^ Cells on both aligned and nonaligned fibers are uniformly distributed, but their morphology varies with fiber orientation. On aligned fibers, cells adopt an elongated shape that aligns with the fiber direction, demonstrating the scaffold’s ability to provide directional cues for cell alignment. In contrast, cells on nonaligned fibers appear more rounded and lack consistent orientation, underscoring the impact of scaffold architecture on cellular behavior. These structural differences suggest the potential of aligned fibers to promote not only higher cell numbers and metabolic activity but also enhanced cell organization and functionality.

### 3.4 Cell Differentiation

Following the evaluation of cell viability and long-term scaffold performance, osteogenic differentiation was conducted to confirm the scaffold’s ability to support healthy cellular behavior. Demonstrating successful differentiation not only verifies the scaffold’s nontoxicity over extended periods but also highlights its capability to promote tissue-specific functions, particularly osteogenic differentiation. This system was specifically selected due to the mechanical and structural properties of electrospun, aligned polystyrene scaffolds, which mimic the native bone extracellular matrix (ECM). Additionally, scaffolds have also been successfully utilized for bone regeneration, supporting the choice of this approach (Table 2). MG63 cells, known for their robust osteogenic potential and consistent differentiation patterns, were selected to reliably evaluate scaffold performance, ensuring clinical relevance compared to traditional tissue culture plastics and alternative scaffold materials.

Alizarin red staining, used to quantify mineral deposition, revealed a significant increase in staining concentration for cells cultured in differentiation media, in day 7, whereas no mineralization was evident in the control (Figure 5A). Notably, the aligned fiber (AF OST) scaffolds outperformed both the nonaligned (No-AF OST) scaffolds and the control, indicating that the architectural cues provided by scaffold alignment play a crucial role in promoting early osteogenesis confirming previous research.^55^

**Figure 5:**
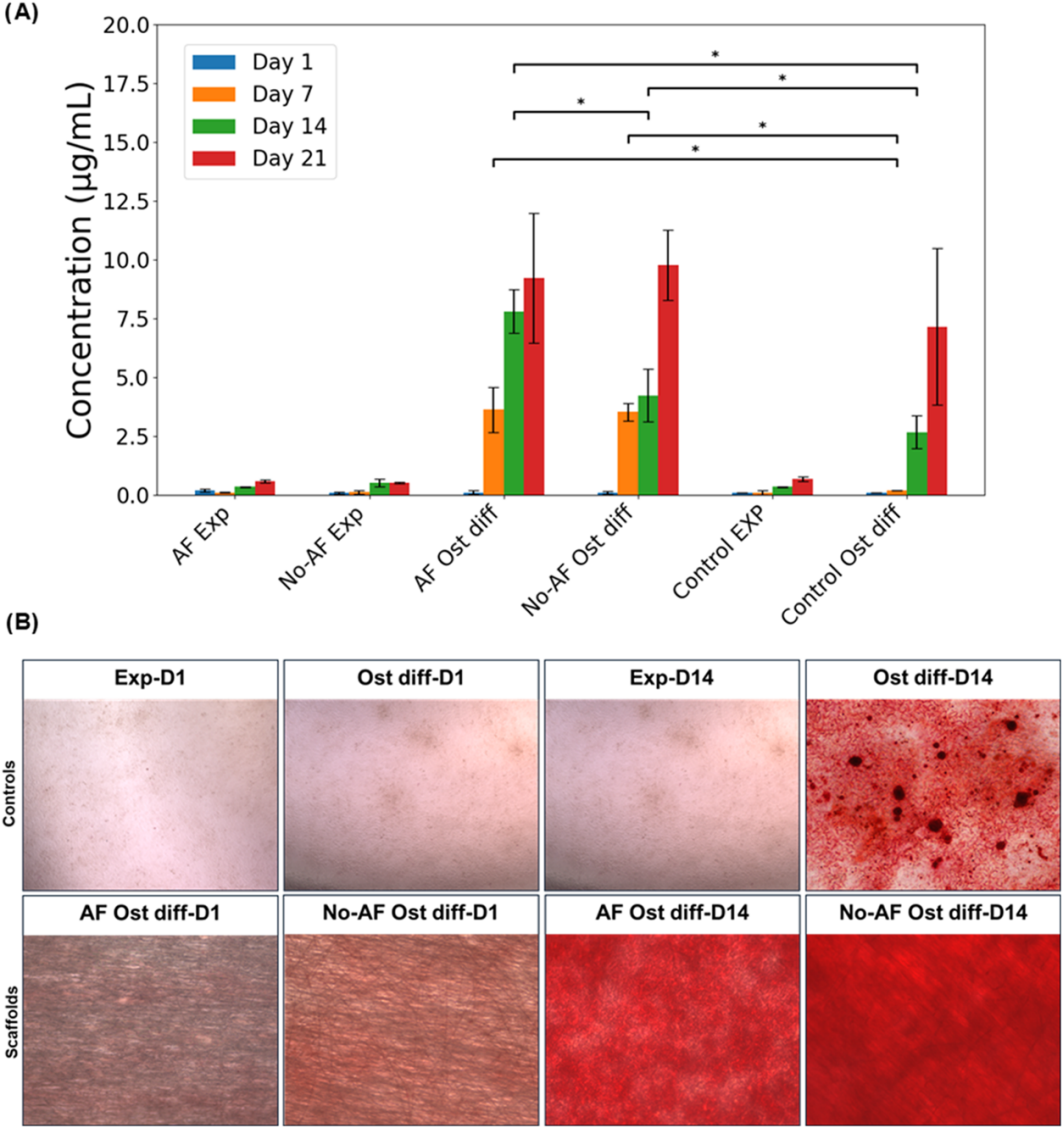
(A) Quantification of the extracted alizarin from the stained sample converted into concentration (μg/mL) across six experimental groups measured at days 1, 7, 14, and 21. Experimental groups include AF Exp (Aligned Fiber Experimental), No-AF Exp (Non-Aligned Fiber Experimental), AF OST diff (Aligned Fiber Osteogenic Differentiation), No-AF Ost diff (Non-Aligned Fiber Osteogenic Differentiation), Control EXP (Control Experimental), and Control Ost diff (Control Osteogenic Differentiation). Error bars represent standard deviations. Statistical significance was assessed using Tukey’s HSD test. Brackets indicate significant differences between pairs of groups (*p* ≤ 0.05), with bracket positions staggered for clarity. (B) Brightfield microscopy of the Alizarin Red staining of undifferentiated and differentiated cells seeded on the scaffold and control at day 1 and day 14.

By day 14, calcium deposition on AF scaffolds was significantly higher than in both the nonaligned and control groups, with this observation persisting through day 21. The calcium level in the Control OST Diff group started to increase on day 14, as expected for typical cell culture plate environment as they are often exhibiting slower osteogenic activity due to fewer biomimetic cues.^56^ In contrast, electrospun scaffolds, characterized by their higher surface area, fiber morphology, and directional cues, support more rapid mineralization.^57^

Bright-field imaging (Figure 5B) shows clear differences in Alizarin Red staining between the scaffold and control conditions. On Day 1, no staining is evident in any group, confirming the absence of mineralization at the experiment’s initiation. By Day 14, prominent Alizarin Red staining is visible in the osteogenic differentiation (OST diff) groups for both the scaffold and control conditions, whereas no staining is observed in the experimental (nondifferentiation) groups. The images highlight the scaffold’s role in supporting localized mineralization compared to the more diffuse staining observed on the control.

These results are further supported and explained by the fact that the fibrous structure of the scaffolds have shown higher cellular metabolic activity compared to the control (Figure 4A). This increased metabolic activity reflects the higher energy demands required for key processes such as cell proliferation, extracellular matrix (ECM) synthesis, and differentiation. The evidence suggests that higher metabolic activity in MG63 cells, as indicated by resazurin-based assays, correlates with earlier mineralization. This relationship is supported by published work linking metabolic engagement with alkaline phosphatase (ALP) activity—a key enzyme involved in matrix formation—and highlighting the importance of metabolic processes in osteoblast differentiation and function.^58,59^ The scaffold’s architecture, particularly the alignment of fibers, provides crucial biophysical signals that stimulate osteoblast activity and support early-stage bone tissue development. These findings underscore the importance of scaffold design in influencing cell behavior and optimizing tissue regeneration.^60,61^ Furthermore, the comparison between DNA concentration (measured by PicoGreen as shown in Figure 4B) and differentiation results highlights the scaffold’s ability to maintain cell viability while supporting differentiation. A plateau in DNA concentration observed between Days 7 and 14 aligns with the onset of differentiation, as cell proliferation typically slows when differentiation begins.

Despite promising outcomes, there is potential for further research in this new approach. Molecular-level analyses, such as gene expression profiling of osteogenic markers including RUNX2 and osteocalcin, performed to offer deeper insights into the mechanisms of differentiation. Additionally, the long-term performance of green-solvent-derived scaffolds, especially their behavior under dynamic conditions, would be of interest to explore. Evaluating these aspects in bioreactor systems will be crucial for tissue engineering scale up research.

Our study highlights the early and elevated metabolic activity and mineralization supported by green solvent electrospun scaffolds, reinforcing their potential as a sustainable alternative to conventional scaffolds. These results make a strong case for their integration into future bone regeneration research. In addition, this study challenges the assumption that polymers such as polystyrene lack utility in tissue engineering, demonstrating that, when processed appropriately, polystyrene-based scaffolds can be highly effective for bone regeneration.

## 4 Conclusions

This work advances sustainable biomaterials by developing a new approach to exploit green solvents in electrospinning to repurpose polystyrene waste from expired laboratory materials into useful functional scaffolds. Eco-friendly solvents dihydrolevoglucosenone (Cyrene) and dimethyl Carbonate (DMC) were able to maintain fiber quality and structural integrity, thereby resulting in smooth, bead-free microfibers with consistent diameters of ca. 2 μm. These dimensions closely mimic the structural features of the bone extracellular matrix, which are essential for supporting cellular adhesion and proliferation. These results demonstrate that our environmentally sustainable methods can produce high-quality scaffolds without compromising material properties. This study confirms that cellular responses align with the structural properties of both aligned and nonaligned fiber scaffolds. Aligned fibers, with strong directional orientation and uniform thickness, enhance MG63 cell activity, making them ideal for tissue engineering requiring organized growth, such as in muscle or bone. Nonaligned fibers supported diverse cell interactions due to the isotropic structure of the scaffold. These interactions demonstrate the potential adaptability for larger-scale, hierarchical tissue engineering scaffolds and confirm the critical role of scaffold architecture in influencing cell behavior and morphology.

While biodegradable materials are often prioritized in scaffold design, nonbiodegradable materials such as polystyrene serve in critical roles where prolonged stability and mechanical integrity are necessary, particularly in long-term cell culture models in vitro and implantable devices that require extended structural support. Upcycling polystyrene into biomaterial scaffolds presents both regulatory and societal challenge, where testing is required to ensure biocompatibility, chemical stability, and safety, making regulatory approval a complex process for upcycled materials. Overcoming these barriers necessitates robust validation, certification from regulatory agencies, and transparent communication about the benefits of upcycled polystyrene. Establishing clear regulatory pathways will therefore play a pivotal role in successfully integrating the findings of this work, to support addressing advances in tissue engineering practices for clinical applications coupled with environmental concerns caused by laboratory waste.

In summary, our new approach to repurpose laboratory materials addresses the dual challenges of plastic waste and the need for innovative biomedical materials by providing a sustainable solution for transforming waste into high-performance scaffolds. Repurposing of discarded plastic into functional biomaterials, not only reduces environmental impact, but also advances tissue engineering practices. This work provides a route for cell culture for tissue engineering related activities to transition toward more sustainable and environmentally conscious scientific practices, thereby aligning with the principles of a circular economy.

## Supplementary Material



## Notes

Annual number of publications on PubMed related to “electrospinning” AND “green solvents” (Figure S1); GPC analysis of purified PS, solubilized PS, green scaffold, and laboratory petri dish for solubilization evaluation (Figure S2); petri dish used as a source polymer for the electrospinning solution (Figure S3); example of image processing and measurement results with threshold adjustments (Figure S4); water contact angle of aligned fibers, non-aligned fiber and plasma treated fibers (Figure S5); 3D model of the custom-designed scaffold holder (Figure S6); Green solvents investigated for the green scaffold electrospun fibers (Table S1); stress vs strain of the aligned fibers samples and non-aligned fibers (Figure S7); DSC characterization of bio-contaminated fibers (Figure S8); thermogravimetric analysis (Figure S9); SEM micrographs of electrospun fibers (Figure S10); effect of polystyrene concentration on fiber morphology (Figure S11); pore size distribution (Figure S12); mechanical properties of aligned and random fibers ultimate tensile strength (Table S2) (PDF)
