# Supplementary material for: Repurposing Laboratory Plastic into Functional Fibrous Scaffolds via Green Electrospinning for Cell Culture and Tissue Engineering Applications
Source: ACS Biomater Sci Eng. 2025 Apr 10;11(6):3573–85. doi: 10.1021/acsbiomaterials.5c00146 (PMC12152829; doi:10.1021/acsbiomaterials.5c00146)
Supplement: Supplementary file 1 [file ab5c00146_si_001.pdf]

Supporting Information

Repurposing Laboratory Plastic into Functional Fibrous Scaffolds via Green Electrospinning for Cell Culture and Tissue Engineering Applications

Nael Berri[1,3], Sandhya Moise[1,3], Antonios Keirouz[1] , Andrew Jennings[2] , Bernardo Castro Dominguez[1,4] Hannah S. Leese[1,3*]

[1]*Department of Chemical Engineering, University of Bath, Bath, BA2 7AY UK*

[2]*Department of Mechanical Engineering, University of Bath, Bath, BA2 7AY UK*

[3]*Centre for Bioengineering and Biomedical Technologies, University of Bath, Bath, BA2 7AY UK (CBio)*

[4]*Centre for Digital Manufacturing and Design, University of Bath, Bath, BA2 7AY UK (dMaDe)*

**Corresponding author* h.s.leese@bath.ac.uk

Key words: *Green solvent, electrospinning, biomaterials, tissue engineering, cell-scaffold interaction, sustainable tissue engineering.*

Table of Contents

Figure S1 Annual number of publications on PubMed related to “electrospinning” AND “green solvents”.

Figure S2 GPC Analysis of purified PS, solubilized PS, green scaffold, and laboratory petri dish for solubilization evaluation.

Figure S3 A) The petri dish used as a source polymer for the electrospinning solution (unprocessed) B) The particles (on the spatula) obtained after milling.

Figure S4 Example of image processing and measurement results with threshold adjustments. Red dots indicate measurement points. (A) and (B) show SEM micrographs of aligned and non-aligned fibers, respectively, while (C) and (D) display the corresponding processed images analyzed using PoreSpy.

Figure S5 Water contact angle of aligned fibers, non-aligned fiber and plasma treated fibers.

Figure S6 3D model of the custom-designed scaffold holder (Autodesk Fusion 360), measuring 15.4 cm in diameter, featuring a visualization window of 8 mm × 10 mm, and including holes of 0.7 mm and 1 mm to allow media flow.

Table S1 Green solvents investigated for the green scaffold electrospun fibers.

Figure S7 Stress vs strain of the aligned fibers samples and non-aligned fibers.

Figure S8 DSC Characterization of bio-contaminated fibers (PS Green scaffold media), clean PS green scaffold, and bulk material (Pellet and petri dish).

Figure S9 Thermogravimetric Analysis (TGA) of cell media-exposed electrospun green fibers measurements was performed using a Setaram Setsys Evo 16/18.0 instrument under an argon atmosphere (100 mL/min).

Figure S10 SEM micrographs of electrospun fibers: (A, B) from petri dishes exposed to cell media for 4 Days and (C, D) from petri dishes not exposed to media; A and B, and C and D, represent different magnifications of the same sample (X500 and X1000).

Figure S11 Effect of polystyrene concentration on fiber morphology: displaying bead formation at A) PS 6 wt% a B) PS 12.5 wt% and C) 15 wt %.

Figure S12 Pore size distribution of A) Non-aligned and B) Aligned fibers scaffold.

Table S2 Ultimate tensile strength (UTS), maximum strain, and Young's modulus for aligned and non-aligned

Supporting figures and tables

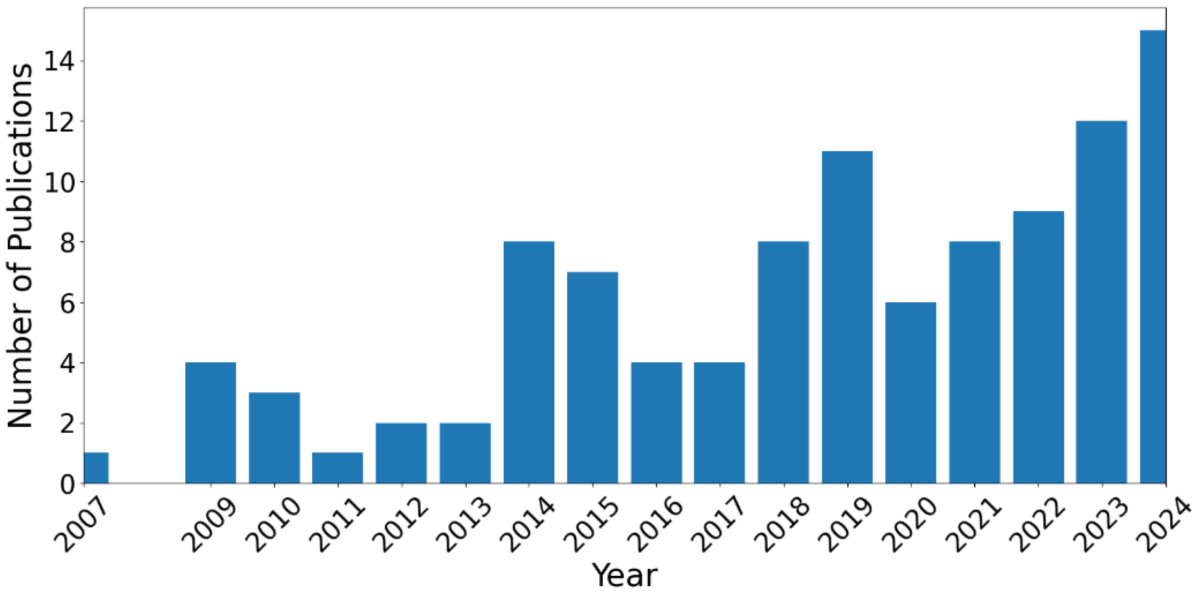

Figure S1 Annual number of publications on PubMed related to “electrospinning” AND “green solvents”.

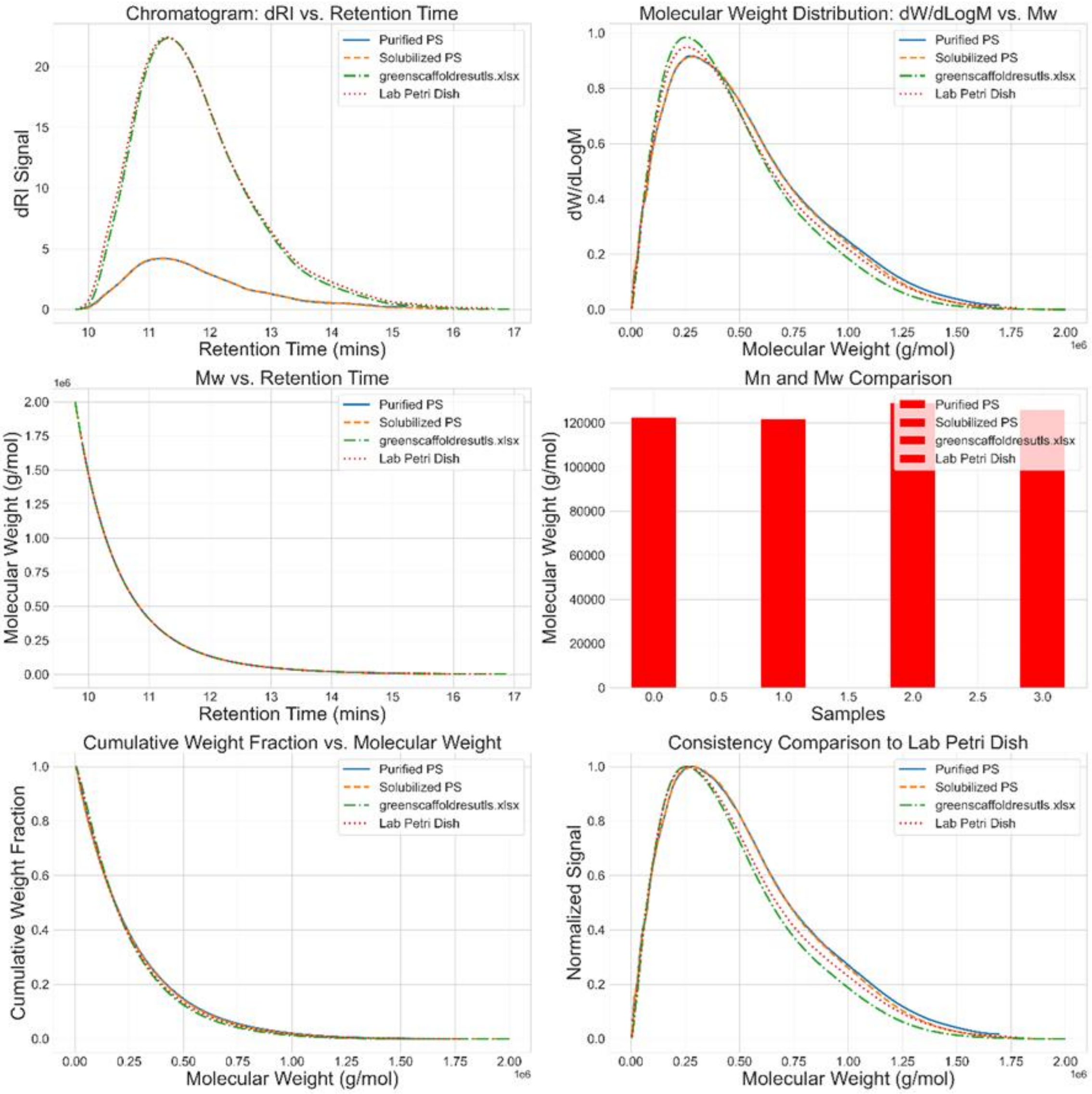

Figure S2 GPC Analysis of purified PS, solubilized PS, green scaffold, and laboratory petri dish for solubilization evaluation.

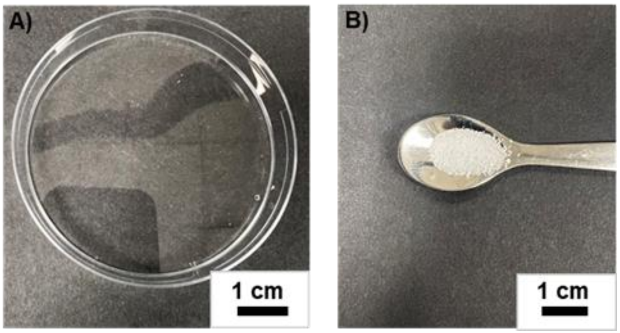

Figure S3 A) The petri dish used as a source polymer for the electrospinning solution (unprocessed) B) The particles (on the spatula) obtained after milling.

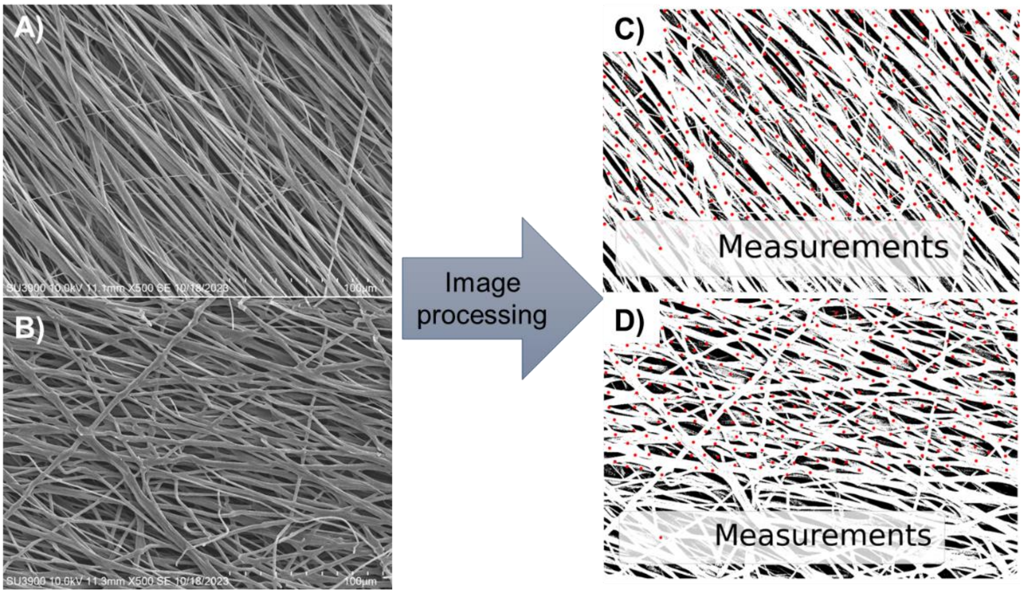

Figure S4 Example of image processing and measurement results with threshold adjustments. Red dots indicate measurement points. (A) and (B) show SEM micrographs of aligned and non-aligned fibers, respectively, while (C) and (D) display the corresponding processed images analyzed using PoreSpy.

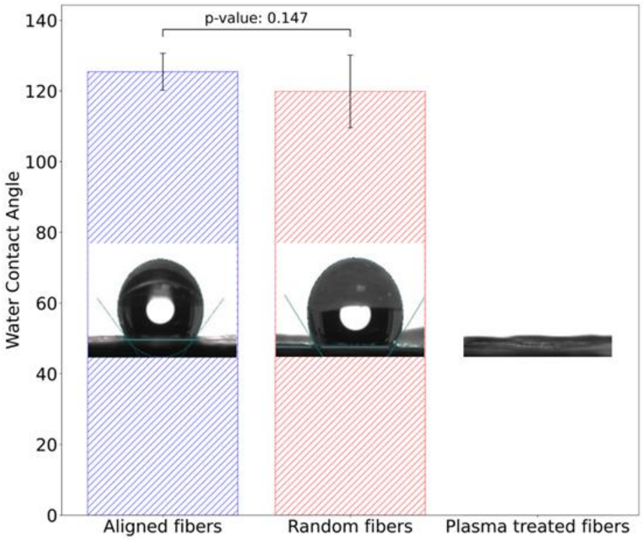

Figure S5 Water contact angle of aligned fibers, non-aligned fiber and plasma treated fibers.

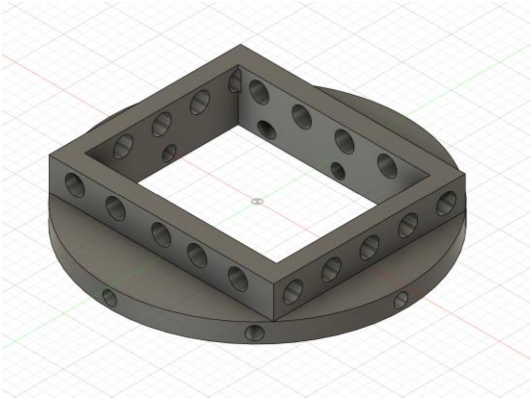

Figure S6 3D model of the custom-designed scaffold holder (Autodesk Fusion 360), measuring 15.4 cm in diameter, featuring a visualization window of 8 mm × 10 mm, and including holes of 0.7 mm and 1 mm to allow media flow.

Table S1 Green solvents investigated for the green scaffold electrospun fibers

Solvent Name	Chemical Formula
Dihydrolevoglucosenone (Cyrene™)	$C_6H_8O_3$
Triethyl Phosphate	$C_6H_{15}O_4P$
Methyl L-lactate	$C_4H_8O_3$
Ethyl L-lactate	$C_5H_{10}O_3$
Glycerol Triacetate (Triacetin)	$C_9H_{14}O_6$
γ-Butyrolactone	$C_4H_6O_2$
Dimethyl Sulfoxide (DMSO)	C_2H_6OS
Acetone	C_3H_6O
Ethanol (EtOH)	C_2H_6O
2-Propanol (Isopropanol)	C_3H_8O
Acetic Acid	$C_2H_4O_2$
Dimethyl Carbonate (DMC)	$C_3H_6O_3$
2-Methyltetrahydrofuran (2-MeTHF)	$C_5H_{10}O$
Dimethyl Isosorbide (DMI)	$C_8H_{14}O_4$

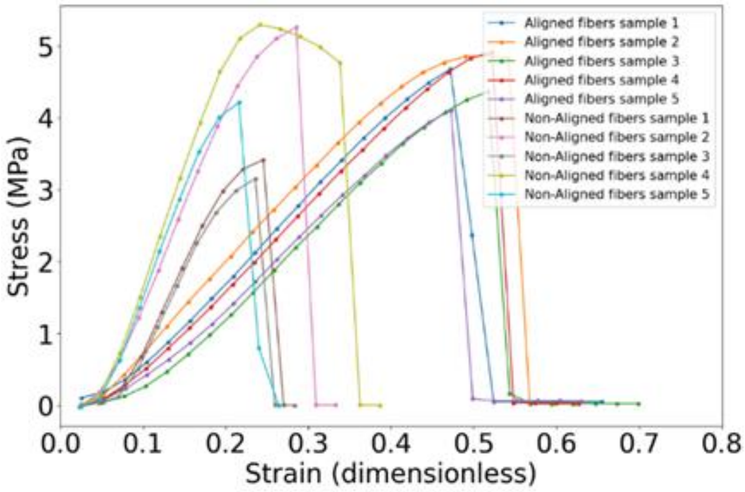

Figure S7 Stress vs strain of the aligned fibers samples and non-aligned fibers.

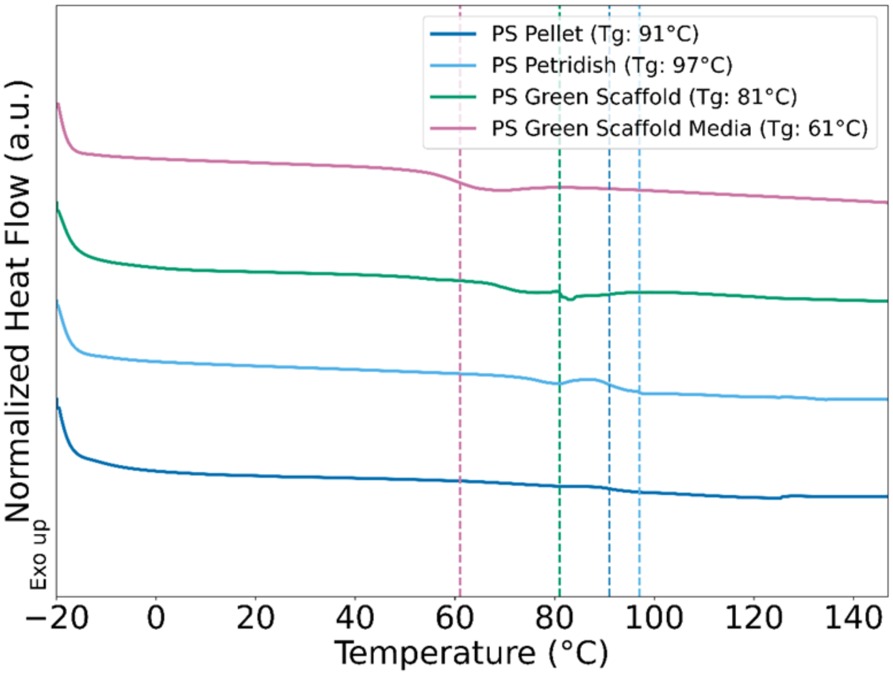

Figure S8 DSC Characterization of bio-contaminated fibers (PS Green scaffold media), clean PS green scaffold, and bulk material (Pellet and petri dish).

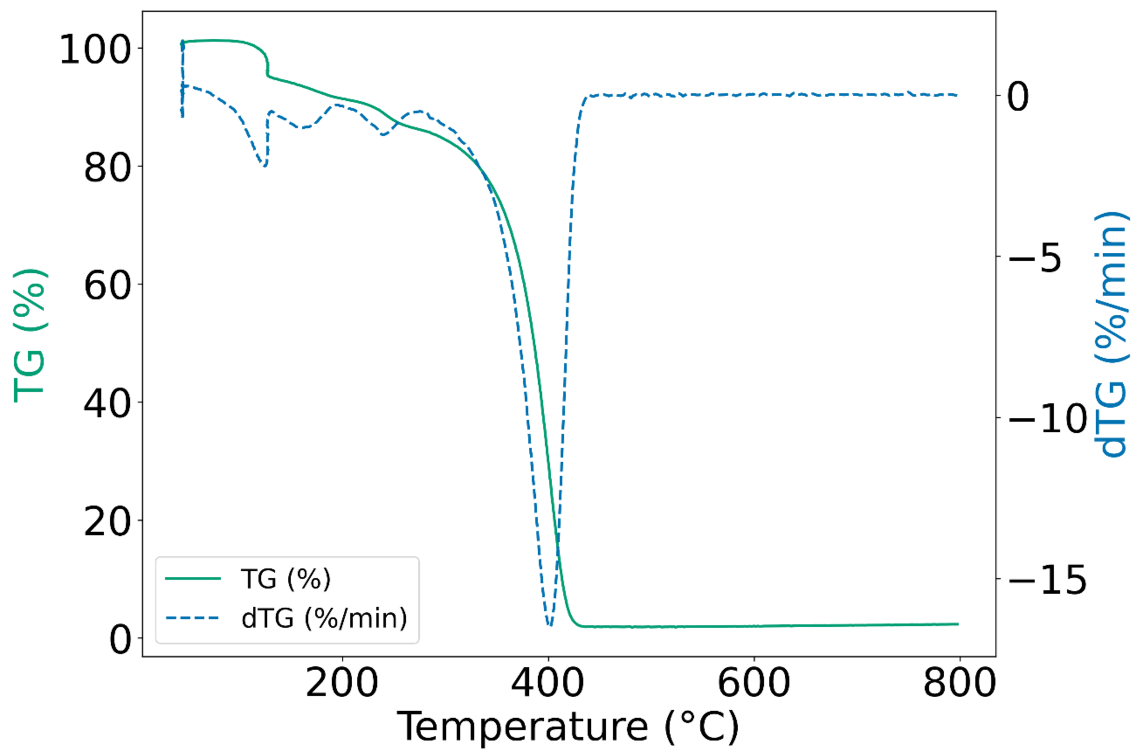

Figure S9 Thermogravimetric Analysis (TGA) of cell media-exposed electrospun green fibers measurements was performed using a Setaram Setsys Evo 16/18.0 instrument under an argon atmosphere (100 mL/min).

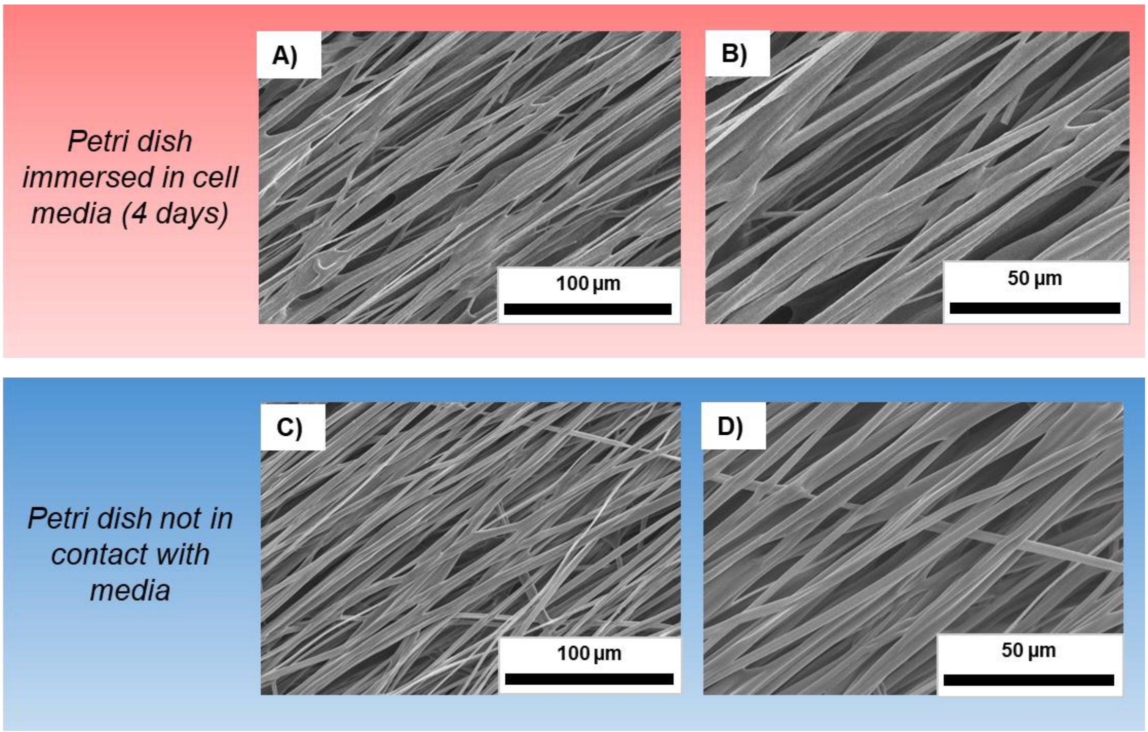

Figure S10 SEM micrographs of electrospun fibers: (A, B) from petri dishes exposed to cell media for 4 Days and (C, D) from petri dishes not exposed to media; A and B, and C and D, represent different magnifications of the same sample (X500 and X1000).

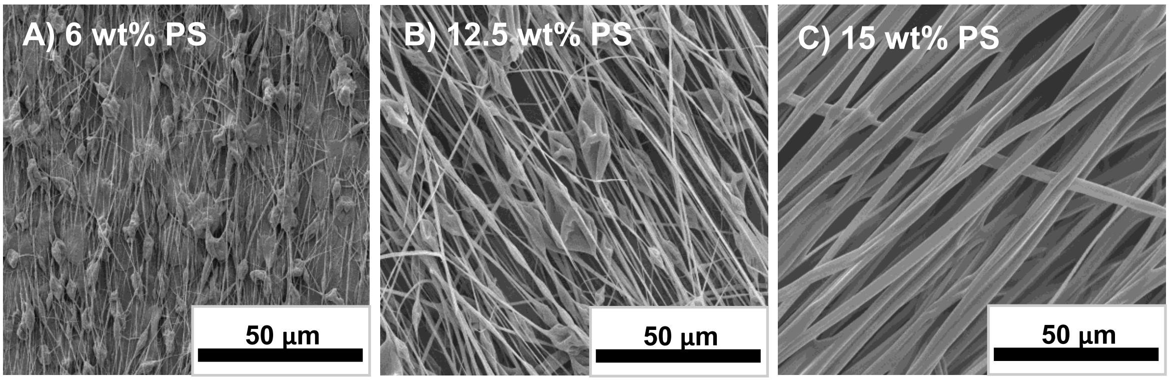

Figure S11 Effect of polystyrene concentration on fiber morphology: displaying bead formation at A) PS 6 wt% a B) PS 12.5 wt% and C) 15 wt %.

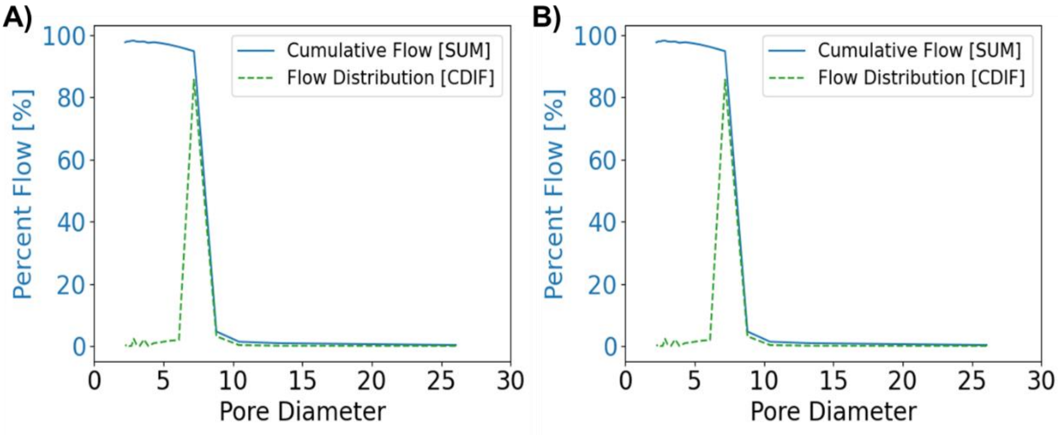

Figure S12 Pore size distribution of A) Non-aligned and B) Aligned fibers scaffold.

Table S2 Ultimate tensile strength (UTS), maximum strain, and Young's modulus for aligned and non-aligned fibers.

Sample type	Replicate	UTS (MPa)	Max Strain	Young's Modulus (MPa)
Aligned Fibers	1	4.68	0.66	12.14
	2	4.87	0.62	12.58
	3	4.36	0.70	11.55
	4	4.91	0.63	11.90
	5	4.11	0.63	11.18
Non-Aligned Fibers	1	3.42	0.27	18.07
	2	5.26	0.33	24.62
	3	3.16	0.28	17.40
	4	5.29	0.39	27.55

5	4.21	0.26	15.20
